# Decline in AmpC β-lactamase-producing *Escherichia coli* in a Dutch teaching hospital (2013-2016)

**DOI:** 10.1371/journal.pone.0204864

**Published:** 2018-10-01

**Authors:** Evert den Drijver, Jaco J. Verweij, Carlo Verhulst, Stijn Oome, Joke Soer, Ina Willemsen, Eefje J. A. Schrauwen, Marjolein F. Q. Kluytmans—van den Bergh, Jan A. J. W. Kluytmans

**Affiliations:** 1 Laboratory for Microbiology and Infection Control, Amphia Hospital, Breda, The Netherlands; 2 Laboratory for Medical Microbiology and Immunology, Elisabeth-TweeSteden Hospital, Tilburg, The Netherlands; 3 Avans Academy for Technology of Health & Environment, AVANS University of Applied Sciences, Breda, The Netherlands; 4 Amphia Academy Infectious Disease Foundation, Amphia Hospital, Breda, The Netherlands; 5 Julius Center for Health Sciences and Primary Care, UMC Utrecht, Utrecht University, Utrecht, the Netherlands; Ross University School of Veterinary Medicine, SAINT KITTS AND NEVIS

## Abstract

**Objective:**

The objective of this study is to determine the prevalence of rectal carriage of plasmid- and chromosome-encoded AmpC β-lactamase-producing *Escherichia coli* and *Klebsiella* spp. in patients in a Dutch teaching hospital between 2013 and 2016.

**Methods:**

Between 2013 and 2016, hospital-wide yearly prevalence surveys were performed to determine the prevalence of AmpC β-lactamase-producing *E*. *coli* and *Klebsiella* spp. rectal carriage. Rectal swabs were taken and cultured using an enrichment broth and selective agar plates. All *E*. *coli* and *Klebsiella* spp. isolates were screened for production of AmpC β-lactamase using phenotypic confirmation tests and for the presence of plasmid-encoded AmpC (pAmpC) genes. *E*. *coli* isolates were screened for chromosome-encoded AmpC (cAmpC) promoter/attenuator alterations.

**Results:**

Fifty (2.4%) of 2,126 evaluable patients were identified as rectal carrier of AmpC β-lactamase-producing *E*. *coli*. No carriage of AmpC β-lactamase producing *Klebsiella* spp. was found. Nineteen (0.9%) patients harboured isolates with pAmpC genes and 30 (1,4%) patients harboured isolates with cAmpC promoter/attenuator alterations associated with AmpC β-lactamase overproduction. For one isolate, no pAmpC genes or cAmpC promotor/attenuator alterations could be identified. During the study period, a statistically significant decline in the prevalence of rectal carriage with *E*. *coli* with cAmpC promotor/attenuator alterations was found (p = 0.012). The prevalence of pAmpC remained stable over the years.

**Conclusions:**

The prevalence of rectal carriage of AmpC-producing *E*. *coli* and *Klebsiella* spp. in patients in Dutch hospitals is low and a declining trend was observed for *E*. *coli* with cAmpC promotor/attenuator alterations.

## Introduction

Antibiotic resistance caused by broad-spectrum β-lactamase production in Gram-negative bacteria is a well-known problem in clinical settings and in the community. Extended-spectrum β-lactamases (ESBL) in Enterobacteriaceae are generally accepted as a major cause of β-lactam resistance [1–3]. Willemsen et al studied the epidemiology of ESBL rectal carriage between 2010 and 2014 in the same teaching hospital. Although the annual prevalence of ESBL was stable, a decline was seen in the proportion of certain ESBL groups, mainly CTX-M-1-1. In addition to ESBL, AmpC β-lactamases are increasingly recognized as a growing and clinically relevant problem [4–8]. Most studies have focused on the dissemination of mobile genetic elements encoding these β-lactamases [5,6,8]. However, in certain species (e.g. *Escherichia coli* and *Shigella* spp.), AmpC β-lactamase production is not only plasmid-encoded, but can also be caused by chromosomal hyperproduction due to mutations within the promoter/attenuator region [4,9,10]. However, little is known on the carriage of either plasmid-encoded (pAmpC) or chromosome-encoded AmpC (cAmpC) Enterobacteriales in hospitalised patients in the North-Western European region and no studies have been performed over a multiple year period. Moreover, screening methods for ESBL, such as ESBL selective media, may not always be optimal to screen for AmpC-producing Enterobacteriales. The present study describes the prevalence of rectal carriage with AmpC β-lactamase-producing *E*. *coli* and *Klebsiella* spp. in patients in Dutch hospitals during a four year period.

## Materials and methods

### Sample collection and phenotypical AmpC testing

Four yearly point prevalence surveys (PPS) were performed in the Amphia Hospital from 2013 to 2016 in the months October or November. All hospitalised patients, including patients on dialysis and day-care, were screened for AmpC carriage using rectal swabs (Eswab, Copan, Italy). After vortexing, the swab was plated on Blood Agar plate (growth control, performed since 2011) and the liquid Amies eluent was inoculated in selective tryptic soy broth, containing cefotaxime (0.25 mg/L) and vancomycin (8 mg/L) (TSB-VC) and incubated for 18–24 hours (35–37°C). In 2013, broths were subcultured on a MacConkey agar containing cefotaxime 1 mg/L (Mediaproducts, Groningen). In 2014, a switch to a more selective double MacConkey agar plate (containing on one side cefotaxime 1 mg/L, cefoxitin 8 mg/L and on the other side ceftazidime 1 mg/L, cefoxitin 8 mg/L, Mediaproducts, Groningen, the Netherlands)was made to improve sensitivity and specificity of the screening. Broth were simultaneously subcultered on both sides of an EbSA agar plate (AlphaOmega, 's-Gravenhage, Netherlands). The Extended Beta-Lactamase Screening Agar (EbSA) plate consists of a split MacConkey agar plate containing ceftazidime (1.0 mg/L) on one side and cefotaxime (1.0 mg/L) on the other side. Both sides contain cloxacillin (400 mg/L) and vancomycin (64 mg/L) for inhibition of AmpC beta-lactamase-producing bacteria and Gram-positive bacteria, respectively. Subsequently, the plates were incubated for 18–24 hours (35–37°C). AmpC producing isolates found in 2013, were retrospectively cultured on the new selective AmpC agar to confirm if they would have been detected using the new agar plate.

For all oxidase-negative isolates that grew on either side of the selective agar plates, species identification was performed by MALDI-TOF (bioMérieux, Marcy l’Etoile, France). The presence of AmpC in all *E*. *coli* and *Klebsiella* spp. isolates was phenotypically confirmed using the D68C AmpC & ESBL Detection Set (Mastdiscs, Mastgroup Ltd, Bootle United Kingdom) and interpreted according to manufacturer's instructions [11,12]. The presence of ESBL in isolates with a MIC of > 1 mg/L for ceftazidime and/or cefotaxime was phenotypically confirmed with the combination disk diffusion method for cefotaxime, ceftazidime, and cefepime with and without clavulanic acid (Rosco, Taastrup, Denmark)) and interpreted according to manufacturer's instructions. Minimal inhibitory concentration (MIC) values for cefotaxime (CTX), ceftazidime (CAZ) and cefoxitin (FOX) were measured using the gradient on a strip method (E-test, bioMérieux, Marcy l'Etoile, France).

### Genetic confirmation of phenotypically confirmed isolates

All phenotypically confirmed *E*. *coli* and *Klebsiella* spp. isolates were screened for the presence of pAmpC genes using the microarray check MDR CT103 according to the manufacturer’s instructions (Check-Points,Wageningen, the Netherlands) [13]. In addition, all phenotypically confirmed *E*. *coli* isolates were subjected to Sanger sequencing of the promoter/attenuator region of the cAmpC gene using M-13 tailed primers as described by Corvec *et al* [14]. The obtained sequences of each isolate were assembled and aligned against the promoter/attenuator region of the cAmpC gene of the *E*. *coli* K-12 strain MG1655 (GenBank database accession number U00096) using Vector NTI Advance 11 software (ThermoFisher Scientific, Waltham, USA) or CLC Genomic Workbench version 8.5 (CLC Bio, Qiagen, Hilden, Germany). Hyperproduction of chromosome-encoded AmpC was assumed when similar alterations in the promoter/attenuator region were found as described by Tracz et al [15].

### Amplified Fragment Length Polymorphism

Isolates from patients who were admitted to the same ward and revealed the same AmpC mechanism were selected for Amplified Fragment Length Polymorphism (AFLP) to determine clonal relatedness. If a patient harboured more than one AmpC-producing isolate from different genus or species, or with different resistance mechanisms or genes, all isolates were typed. AFLP typing was performed and interpreted as described by Savelkoul *et al* [16].

### Statistical methods

All data were pseudonymised and subsequently analysed with Statistical Package for Social Science software (SPSS; IBM Corp., Armonk, New York, US; version 19). The 95% confidence intervals of proportions were calculated using the modified Wald method. A trend analyses for the prevalence of AmpC genes was performed using the Mantel-Haenzsel test for linear association. The trend result was adjusted for gender using logistic regression. Statistical significance was accepted at p <0.05.

### Ethical considerations

The yearly PPS for AmpC-producing *E*. *coli* and *Klebsiella* spp. rectal carriage is part of the routine hospital infection control policy and is approved by the management of the hospital. This is in accordance with the current regulations in the Netherlands and requires verbal consent from participating patients. According to the Dutch regulation for research with human subjects, neither medical nor ethical approval, was required to conduct the surveillance since it was part of the local hospital policy, and all data were processed anonymously. Patient who indicated that they did not want to participate in the survey were excluded (opt-out)[3].

According to the patient’s preferences, samples were taken by nursing staff or by patients themselves. The Infection Control Practitioner collected the information on the patient characteristics from the medical record.

## Results

A total of 2,527 patients were eligible for AmpC screening. Response rates over the four-year period are shown in Table 1. A total of 2,126 evaluable cultures (positive growth control) were collected and screened for the presence of AmpC rectal carriage. The median age of screened patients was 66 years (range 0–100 years) and 52% of the patients were female. The median length of hospital stay was 2 (range 0–79) days. In total 1,783 (84%) patients were admitted to a clinical ward, 343 (16%) were considered as day-care. The distribution of patients across various medical specialties is shown in Table 1. Over the four year period this distribution remained stable, with the majority of patients in internal medicine (25%) and general surgery (17%) wards.

**Table 1:** Overview of the hospitalized patients on the days of survey, cultured patients, negative growth control, response rate, evaluable cultures, as well as baseline characteristics of screened patients in point prevalence surveys over the years (2013–2016).

	2013	2014	2015	2016	Total
**Hospitalized patients on day of survey (no.)**	601	652	654	620	2,527
**Response rate (%)**	85.9%	87.3%	86.2%	83.1%	85.6%
**No rectal swab taken**	85	83	90	105	363
**Negative growth control**	8	12	4	15	39
**Patients with evaluable cultures (%)**	508 (84.5%)	557 (85.4%)	560 (85.6%)	501 (80.8%)	2,126 (84.1%)
Age in years, median (range)*	65(0–93)	65(0–99)	67(0–100)	67(0–97)	66 (0–100)
Male, no. (%)*	259(51%)	268(48%)	269(48%)	231(46%)	1,027 (48%)
Hospitalization>2days, no. (%)*	231(46%)	271(49%)	222(40%)	269(54%)	993 (39%)
Length of stay on day of culture, median in days, (range)*	2(0–51)	2(0–70)	2(0–47)	3(0–79)	2 (0–79)
Patients in day-care, no. (%)*	66(13%)	101(18%)	114(20%)	62(12%)	343 (16%)
Medical specialty, no. (%)*					
**Anesthesiology (non-ICU)**	10 (2%)	9(2%)	23 (4%)	14(3%)	56 (3%)
**Cardiology**	46(9%)	50(9%)	47(8%)	51(10%)	194 (9%)
**Geriatrics**	11(2%)	11(2%)	9(2%)	12(2%)	43 (2%)
**Intensive Care Unit (ICU)**	17(3%)	14(3%)	14(3%)	20(4%)	65 (3%)
**Internal medicine**	132(26%)	134(24%)	131(23%)	126(25%)	523 (25%)
**Neurology**	31(6%)	35(6%)	29(5%)	32(6%)	127 (6%)
**Obstetrics and gynaecology**	43(8%)	21(4%)	40(7%)	20(4%)	124 (6%)
**Orthopedic surgery**	34(7%)	42(8%)	37(7%)	40(8%)	153 (7%)
**Otorhinolaryngology**	8(2%)	20(4%)	14(3%)	10(2%)	52 (2%)
**Pediatrics**	24(5%)	31(6%)	18(3%)	27(5%)	100 (5%)
**Pulmonary diseases**	32(6%)	41(7%)	43(8%)	33(7%)	149 (7%)
**Surgery, cardiothoracic**	17(3%)	17(3%)	24(4%)	17(3%)	75 (4%)
**Surgery, general**	86(17%)	94(17%)	93(17%)	89(18%)	362 (17%)
**Urology**	17(3%)	21(4%)	18(3%)	8(2%)	64 (3%)
**Other speciality**	0(0%)	17(3%)	20(4%)	2(0%)	39 (2%)

*Data and percentages based upon evaluable cultures

The prevalence of AmpC rectal carriage over the four-year period is shown in Table 2. All AmpC-producing isolates were identified as *E*. *coli*. No AmpC producing *Klebsiella* spp were found. Eighteen of 19 (94.7%) of the pAmpC-producing isolates harboured *bla*_CMY-2-like,_ one isolate *bla*_DHA-like_. The prevalence of 3^rd^ generation cephalosporine resistant *E*.*coli*, based on growth on selective media in the evaluable cultures, and the amount of ESBL confirmed isolates are shown in Table 2 as well.

**Table 2:** Prevalence of patients with AmpC-producing E. coli and distribution of pAmpC genes and cAmpC hyperproducers over the years.

	2013	2014	2015	2016	Overall
**Total *E*.*coli* cultured on selective media (no. and %)^1^**	43 (8.5%)	48 (8.6%)	41 (7.3%)	20 (3.9%)	152 (7.1%)
**Total AmpC isolates (no. and %)^2^**	19(3.7%)	16(2.9%)	9(1.6%)	6(1.2%)	50(2.4%)
**Primary (no.)**	18	14	9	6	47
**Secondary (no.)^3^**	1	2	0	0	3
**AmpC genes (incl. secondary cases)**					
***bla*_CMY-2-like_**	7	6	2	3	18
***bla*_DHA-like_**	0	0	0	1	1
**cAmpC hyperproducers**	11	10	7	2	31
**Inconclusive^4^**	1				
**Total ESBL isolates (no. and %)^5^**	25 (4.9%)	29 (5.2%)	32 (5.7%)	13 (2.6%)	99 (4.7%)

1 = Number of patients with *E*. *coli* rectal carriage based on selective media divided by the number of patients with evaluable cultures

2 = Number of patients with AmpC-producing *E*. *coli* rectal carriage divided by the number of patients with evaluable cultures

3 = Number of patients with isolates that were clonally related based on AFLP

4 = negative microarray results for pAmpC genes, but AmpC amplicon couldn’t be amplified

5 = Number of patients with ESBL-producing *E*. *coli* rectal carriage divided by the number of patients with evaluable cultures

All but one (30 out of 31) of the isolates that were phenotypically AmpC producers and negative for pAmpC in the microarray, showed alterations in the promoter/attenuator region associated with cAmpC hyperproduction [15]. Moreover, none of the pAmpC producing isolates (n = 19) showed mutations that are associated with cAmpC hyperproduction.

One isolate could not be amplified using Sanger sequencing technique and was negative in Micro-array which was therefore considered as inconclusive. An overview of all alterations in the promoter/attenuator region as well as MIC values for cefotaxime, ceftazidime and cefoxitin can be found in the additional S1 Table.

In 2013, two *bla*_*CMY-2-like*_ producing *E*. *coli* isolates from two patients were confirmed to have the same AFLP pattern. Three cAmpC producing *E*. *coli* (all Mulvey Type 3) from three different patients in 2014 showed a similar AFLP pattern as well. AFLP patterns can be found in the additional data S1 Fig.

**Fig 1:**
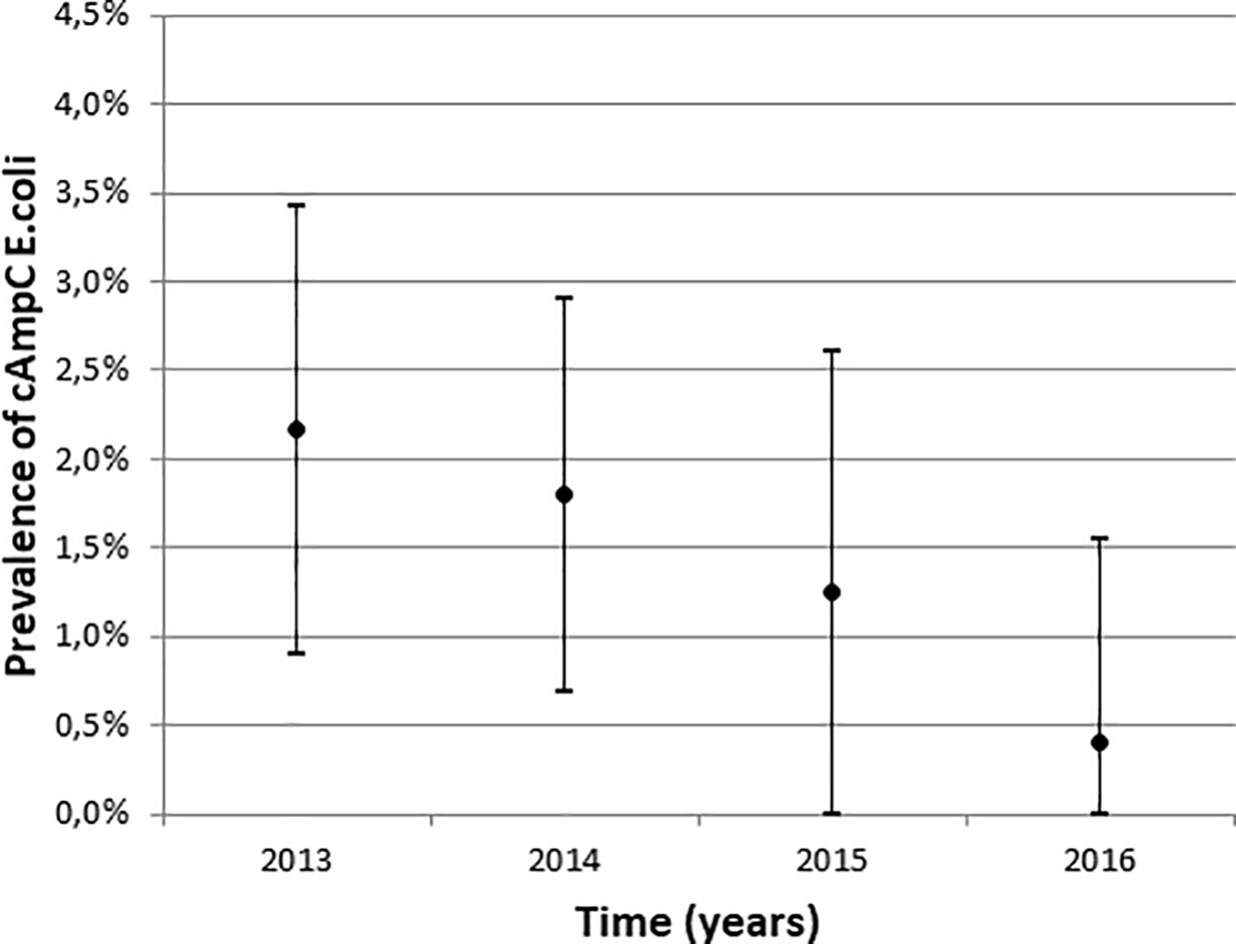
Prevalence of cAmpC hyperproducing E. coli from 2013 to 2016 with 95% confidence interval.

**Fig 2:**
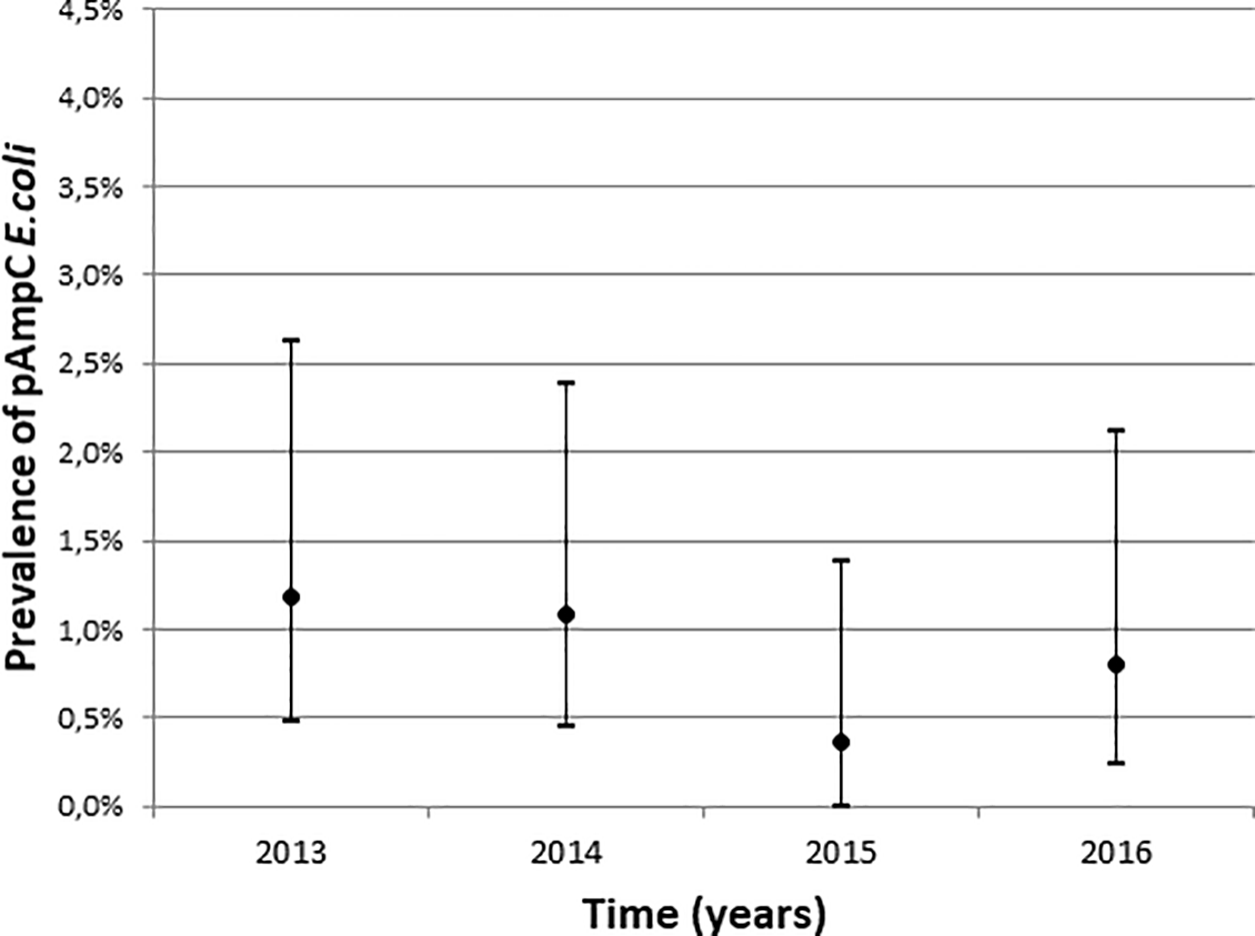
Prevalence of pAmpC producing E. coli from 2013 to 2016 with 95% confidence interval.

A declining trend was seen in the overall prevalence of AmpC in *E*. *coli* isolates using Mantel-Haenzsel test for linear association (p = 0.006). When probable genetically related AmpC isolates based upon AFLP were excluded the decline was still significant. The decrease was only significant for cAmpC hyperproducers (p = 0.012) and not for pAmpC (p = 0.287) (Figs 1 and 2). A univariable and multivariable logistic regression analysis was performed on the cAmpC rectal carriage prevalence over the four-year period (2013–2016) adjusted for gender (univariate analysis, p = 0.15) (see supplementary data, S2 Table).

## Discussion

The present study shows a significant decline of the prevalence of cAmpC producing *E*. *coli* measured in four yearly point prevalence surveys. The pAmpC prevalence remained stable over time. To the best of our knowledge, there are no other studies published on trends in rectal carriage of both cAmpC and pAmpC positive isolates in hospitalised patients, which prohibits a comparison of trends with other studies.

Few studies have looked at pAmpC carriage in hospitalised patients. Garrido *et al* found a low prevalence of 0.59% pAmpC in faecal sample of a Spanish hospitalised population[17]. In the same year Husickova *et al* published a survey on rectal carriage of pAmpC producing isolates and found a prevalence of 0.3%[18]. These results are slightly lower in comparison to our study. However, both studies did not use a (selective) pre-enrichment broth, which may explain the lower pAmpC prevalence [19]. No studies were performed on the occurrence of rectal carriage of cAmpC isolates in hospitalised patients. Data on the prevalence of AmpC rectal carriage in the community within the North Western European region is more extensively described, although not all studies screened or confirmed cAmpC production in *E*. *coli isolates*. In Norway, Ulstad *et al*[20] found a carriage rate of 3.2% of phenotypically confirmed AmpC-producing *E*.*coli* and 0.7% pAmpC producers based on PCR and WGS. In the Netherlands three studies on AmpC prevalence were performed in the community. A random sample of adult volunteers (n = 1033) by van Hoek *et al* [21] found an overall AmpC prevalence of 1.4%, consisting of 0.6% pAmpC and 0.8% cAmpC. Koningstein *et al* [22] screened faecal samples of day-care centre attending children and found an AmpC rectal carriage rate of 2.4%, with 1.2% *bla*_CMY-2_ producing *E*.*coli* and 1.1% cAmpC hyperproducers [23]. Finally, a study by Reuland *et al* [24], that screened adult volunteers in the region of Amsterdam found 1.3% carriage of pAmpC producing *E*. *coli*. These results correspond with the prevalence rates in the present study for 2013 and 2014 for pAmpC and cAmpC. The three Dutch studies used pre-enrichment broths, but applied different screening tests. No studies have looked at trends over time in rectal carriage of AmpC. To our knowledge this study is the first to show a declining trend of cAmpC within the North-Western European region.

Based on the mentioned criteria in combination with AFLP, few cases of probable genetic clonality were detected. As not all isolated were subjected to strain typing a conclusion on epidemiological relationship cannot be made. Although we noticed heterogeneity of cAmpC alterations over the years, we cannot fully rule out the possibility of clustering of cAmpC isolates during first years in the strains that were not subjected to AFLP. Future analysis is needed to clarify the situation of clonal spread and the possibility of horizontal spread of resistance genes.

Trend analysis was adjusted for gender using logistic regression analysis. Data on other risk factors known for ESBL-E carriage, which might be applicable to AmpC carriage as well, like travel history or antibiotic use before admission were not available.

A limitation of this PPS is the lack of evidence on sensitivity and specificity of screenings agars used to detect AmpC producing isolates. No commercial AmpC specific screenings agars are available. During the 4-year period, a change in our used screenings agar was made to improve the sensitivity and specificity. The used MacConkey agar, containing cefotaxime 1 mg/L (Mediaproducts, Groningen) was accepted to be sensitive for AmpC producing isolates, but not very specific, as other cephalosporin resistant Enterobacteriaceae (e.g. ESBL or K1-betalactamase producing isolates) were cultured as well. This could lead to overgrowth of isolates with other resistance mechanisms. To improve specificity, new AmpC agars were used, containing cefoxitin and cefotaxime as well as a combination of cefoxitin and ceftazidime. Retrospective screening of the 2013 isolates on the new screenings agar showed growth of all AmpC producing isolates. Unfortunately, we were not able to use the original rectal swabs to study the new screening agar.Loss of sensitivity because of the addition of cefoxitin was expected to be negligible, as most AmpC producing isolates described in literature, have increased MIC’s for cefoxitin [4,15,25]. The switch in media is unlikely to be the reason for the decline in AmpC. Using both cefotaxime and ceftazidime might even increase the sensitivity, using two different cephalosporin antibiotics. Only the *bla*_ACC-like_ producing isolates could be of concern, which are known to have lower MIC’s for cefoxitin [25]. However, we did not find any *bla*_ACC-like_ producing isolates with either of the used agars. Still, a comparative study on the diagnostic performance of screenings agars for AmpC producing isolates, might provide more insight on this matter.

Another limitation in our study is the use of a micro-array system. Although we assume that the AmpC phenotype can be related to the microarray- and sanger sequencing data, we cannot discard that micro-array system only recognize the six main plasmid AmpC-like groups (*bla*_CMY-2-like_, *bla*_DHA-like_, *bla*_ACC-like_, *bla*_ACT/MIR-like_, *bla*_FOX-like_, *bla*_CMY-1/MOX-like_). Although former studies showed robust sensitivity and specificity of the microarray check MDR CT103 [13,26,27], it is limited to these specific targets.

We did not perform reverse-transcriptase PCR, as described by Trazc et al [15]. Nevertheless, we expect that hyperproduction of AmpC is likely, when known alterations in promoter and attenuator region were found as described in this paper.

It is difficult to ascertain the cause of the decrease in cAmpC during the four-year period. The link between trends in e.g. ESBL and pAmpC and the dissemination of these β-lactamases in the environment (e.g. food products, livestock, companion animals), as well as the influence of antibiotic usage in both humans and livestock, is under debate. Carriage of cAmpC has been described in studies on veal calves [28], broilers [29] and companion animals [30,31] within the Netherlands. The study by Hordijk *et al* showed an increase of cAmpC in veal cattle in the period 1997 to 2010 [28]. Between 2009 to 2015 a decrease of 58.4% of antibiotic sales in the livestock sector was achieved[32]. However, to our knowledge, no new data on cAmpC prevalence in livestock in the Netherlands has been published since the decrease in antibiotic sales. Therefore, we can only speculate on the possible impact of the antibiotic decrease in the veterinary sector and the decreasing trend we found.

## Conclusions

Four yearly point prevalence surveys show that rectal carriage of both pAmpC and cAmpC beta-lactamase producing *E*. *coli* and *Klebsiella* spp. was low. This study shows a significant decline in rectal carriage of *E*. *coli* with cAmpC promotor/attenuator alterations in hospitalised patients in a Dutch teaching hospital during this four year period. The underlying reasons are unclear and deserve further investigations as they may provide new insights to further control antimicrobial resistance.

## Supporting information

S1 Table Overview of AmpC promoter/attenuator alterations of all AmpC producing *E. coli*, as well as MIC values for cefotaxime, ceftazidime and cefoxitin. (PDF)

S2 Table Univariable and multivariable logistic regression analysis of cAmpC carriage during a four-year period (2013–2016) adjusted for gender. CI confidence interval.
(DOCX)

S1 Fig AFLP patterns from all AmpC producing *E. coli* eligible for cluster analyses (2013 & 2014). (DOCX)
