# Supplementary material for: Decline in AmpC β-lactamase-producing Escherichia coli in a Dutch teaching hospital (2013-2016)
Source: PLoS One. 2018 Oct 1;13(10):e0204864. doi: 10.1371/journal.pone.0204864 (PMC6166941; doi:10.1371/journal.pone.0204864)
Supplement: S1 Table — (PDF) [file pone.0204864.s001.pdf]

Strain	Acquired β-lactamase	Mulvey type‡	-118	-88	-82	-73	-42	-32	-28	-18	-16	-15	-13	-11	-1	6	17	22	24	31	33	34	35	37	58	63	70	81	cAmpC	E-test		
E.coli K12			T	C	A	C	C	T	G	G	–	–	–	C	C	C	C	C	C	C	G	G	C	G	C	T	C	G	Hyperproduction†	MIC cfxt (mg/L)	MIC cftx (mg/L)	MIC cftz (mg/L)
AmpC2013-A*	none	23				T		A																				A	Yes (19-fold)	32	0,75	1,5
AmpC2013-B	none	n/a																											n/a	24	0,75	1,5
AmpC2013-C*	none	8				T							T			T				A								A	Yes (24-fold)	128	3	2
AmpC2013-D	none	3		T	G		T			A					T										T				Yes (22-fold)	32	1,5	3
AmpC2013-E	none	unknown				T						G													T	C		A	Yes (16-fold)	48	1	1,5
AmpC2013-F	none	unknown				T						G													T	C		A	Yes (16-fold)	32	1	1,5
AmpC2013-G*	none	3		T	G		T			A					T										T				Yes (22-fold)	96	3	6
AmpC2013-H	none	unknown		T	G		T			A					T			A							T				Yes (≈60-140-fold)	96	4	4
AmpC2013-I*	TEM wt	3		T	G		T			A					T										T				Yes (22-fold)	32	12	32
AmpC2013-J	none	3		T	G		T			A					T										T				Yes (22-fold)	24	1,5	2
AmpC2013-K*	TEM wt	unknown	A			T			A			G																A	Yes (16-fold)	64	1,5	2
AmpC2013-L*	none	2				T		A																	T	C		A	Yes (10-fold)	24	0,75	1
AmpC2013-M	CMY-2	11																									T		No	≥256	16	24
AmpC2013-N	CMY-2	12				T																			T	C		A	No	48	6	6
AmpC2013-O*	CMY-2 + TEM wt	12				T																			T	C		A	No	96	8	12
AmpC2013-P*	CMY-2 + TEM wt	12				T																			T	C		A	No	96	8	12
AmpC2013-Q	CMY-2	11																									T		No	96	6	6
AmpC2013-R	CMY-2 + TEM wt	7				T			A								T											A	No	192	12	32
AmpC2013-S*	CMY-2	unknown	A			T																						A	No	96	8	16
AmpC2014-A	none	unknown				T		A	A																			A	Yes (8-fold)	24	0,75	1
AmpC2014-B	none	unknown				T		A																	T	C			Yes (8-fold)	16	0,75	1
AmpC2014-C	none	unknown												T								del					T		Yes ((≈21-68 fold)	48	1,5	1,5
AmpC2014-D	none	3		T	G		T			A					T										T				Yes (22-fold)	12	1,5	2
AmpC2014-E*	none	3		T	G		T			A					T										T				Yes (22-fold)	12	0,75	2
AmpC2014-F*	none	3		T	G		T			A					T										T				Yes (22-fold)	12	0,5	2
AmpC2014-G*	none	3		T	G		T			A					T										T				Yes (22-fold)	16	2	2
AmpC2014-H	none	3		T	G		T			A					T										T				Yes (22-fold)	48	3	4
AmpC2014-I	none	unknown									C									G							T		Yes ((≈16-64 fold)	64	2	3
AmpC2014-J	none	8				T							T			T				A								A	Yes (24-fold)	24	0,75	1
AmpC2014-K	CMY-2 + TEM wt	11																									T		No	24	32	32
AmpC2014-L	CMY-2	11	A			T			A																			A	No	96	12	24
AmpC2014-M	CMY-2	11																									T		No	≥256	32	48
AmpC2014-N	CMY-2+CTX-M-1-15	wildtype																											No	192	≥32	48
AmpC2014-O*	CMY-2	52																									T		No	128	8	24
AmpC2014-P*	CMY-2	11																									T		No	≥256	≥32	48
AmpC2015-A	neg	unknown				T						G																A	Yes (16-fold)	32	1	2
AmpC2015-B	neg	unknown				T							T								A				T	C		A	Yes (24-fold)	32	1	1
AmpC2015-C	neg	3		T	G		T			A					T										T				Yes (22-fold)	64	1,5	3
AmpC2015-D	neg	3		T	G		T			A					T										T				Yes (22-fold)	32	2	4
AmpC2015-E	neg	3		T	G		T			A					T										T				Yes (22-fold)	16	≥32	8
AmpC2015-F	CMY-2	WT																											No	128	8	24
AmpC2015-G	CMY-2	WT																														
AmpC2015-H	neg	3		T	G		T			A					T										T				Yes (22-fold)	≥256	4	6
AmpC2015-I	neg	23				T		A															A						Yes (19-fold)	48	2	2
AmpC2016-A	CMY-2	12				T																			T	C		A	No	≥256	≥32	128
AmpC2016-B	CMY-2 + TEM wt	18		T	G					A					T										T				No	≥256	≥32	48
AmpC2016-C	CMY-2 + TEM wt	11																									T		No	≥256	≥32	48
AmpC2016-D	DHA	unknown																						T			T		No	256	24	24
AmpC2016-E	neg	unknown				T						G																A	Yes (16-fold)	24	1	16
AmpC2016-F	neg	3		T	G		T			A					T										T				Yes (22-fold)	24	0,75	1
							↓ displaced -35 box	-35 box		↓ displaced -10 box				-10 box					attenuator													

Position number as described by Mulvey et al[17]. Positions -42 and -18 are involved in de displaced promoter box, positions -32 and -11 are the wild type promoter boxes. Positions -16, -15 and -13 contain insertions in the spacer region of the promoter. The attenuator region is located between position 17 and position 37.

*Amplification Fragment Length Polymorphism (AFLP) technique performed by the VU Amsterdam

‡ As defined by Mulvey et al. (2005). The reader should refer to Table 3 of the given reference to see a complete characterization of each promoter type.

† Delta–delta cycle threshold, interpreted as fold-expression as defined by Tracz et al. (2007 [16])
