# Supplementary material for: Decline in AmpC β-lactamase-producing Escherichia coli in a Dutch teaching hospital (2013-2016)
Source: PLoS One. 2018 Oct 1;13(10):e0204864. doi: 10.1371/journal.pone.0204864 (PMC6166941; doi:10.1371/journal.pone.0204864)
Supplement: S2 Table — CI confidence interval. (DOCX) [file pone.0204864.s002.docx]

S2 Table Univariable and multivariable logistic regression analysis of cAmpC carriage during a four-year period (2013-2016) adjusted for gender. CI confidence interval.

|  | Univariable logistic regression analysis | | | Multivariable logistic regression analysis | | |
| --- | --- | --- | --- | --- | --- | --- |
|  | **Odds Ratio** | **95% CI** |  | | **Odds Ratio** | **95% CI** |
| 2013 | reference |  |  | | reference |  |
| 2014 | 0.826 | 0.348-1.962 |  | | 0.813 | 0.342-1.932 |
| 2015 | 0.572 | 0.220-1.487 |  | | 0.563 | 0.238-1.463 |
| 2016 | 0.181 | 0.040-0.821 |  | | 0.176 | 0.039-0.800 |
| Gender (male) | 1.628 | 0.771-3.438 |  | | 1.681 | 0.795-3.555 |
| Short stay | 1.037 | 0.394-2.727 |  | |  |  |
| Age, median | 1.004 | 0.988-1.021 |  | |  |  |
