# Supplementary material for: Decline in AmpC β-lactamase-producing Escherichia coli in a Dutch teaching hospital (2013-2016)
Source: PLoS One. 2018 Oct 1;13(10):e0204864. doi: 10.1371/journal.pone.0204864 (PMC6166941; doi:10.1371/journal.pone.0204864)
Supplement: S1 Fig — (DOCX) [file pone.0204864.s003.docx]

S1 Fig AFLP patterns from all AmpC producing *E. coli* eligible for cluster analyses (2013 & 2014)

1. *E.coli* 2013


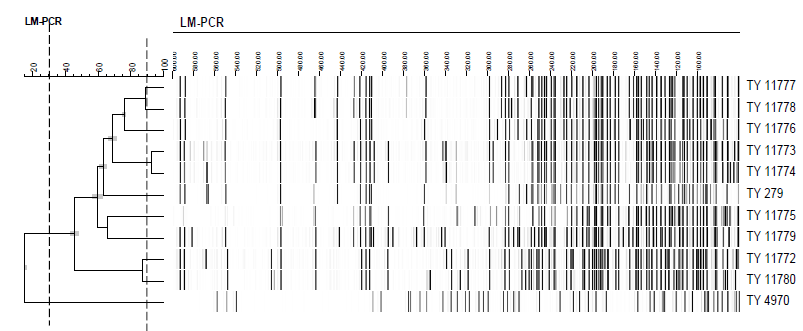


AmpC2013-C

AmpC2013-A

AmpC2013-L

AmpC2013-P

AmpC2013-O

ATCC 35218 *E.coli*

AmpC2013-S

AmpC2013-K

AmpC2013-G

AmpC2013-I

DSM 4564 *K.pneumoniae*

Dds

1. *E.coli* 2014

AmpC2014-P

AmpC2014-O

ESBL2014-A

ATCC 35218 *E.coli*

ESBL2014-B

ESBL2014-C

ESBL2014-D

ESBL2014-F

ESBL2014-G

AmpC2014-E

AmpC2014-G

AmpC2014-F

DSM4564 *K.pneumoniae*


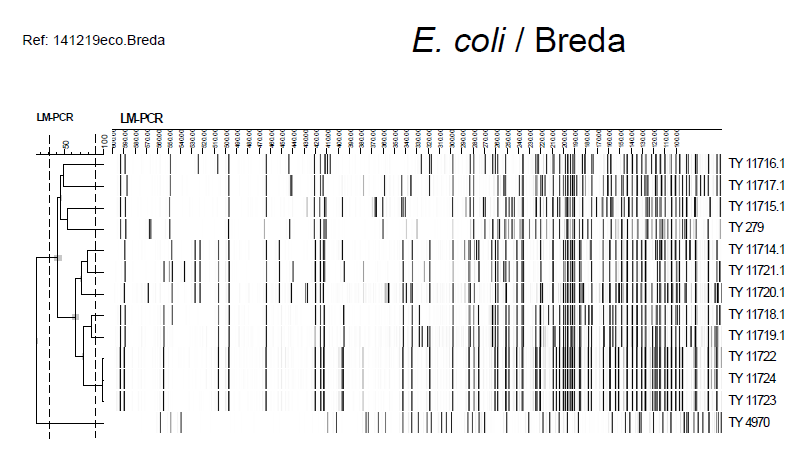


*AFLP patterns from all AmpC producing E. coli eligible for cluster analyses. Strains clustering with a similarity between 90 and 100% were defined as identical strains. Strains clustering with a similarity above 35% were defined as different strains of the same species and strains clustering with a similarity below 35% were defined as different species. Identical strains are indicated in color. Each strain was coded with type of resistance mechanism, the number of the year in combination with a letter. ATCC 35218 E.coli and DSM 4564 K.pneumoniae were used as reference strains. In 2014 a number of E.coli ESBL strains were selected for AFLP typing as well (further data on ESBL strains not shown is this publication).*
